# Toward a Unified Analysis of the Brain Criticality Hypothesis: Reviewing Several Available Tools

**DOI:** 10.3389/fncir.2022.911245

**Published:** 2022-05-20

**Authors:** Chaojun Yu

**Affiliations:** School of Mathematical Sciences, Zhejiang University, Hangzhou, China

**Keywords:** brain criticality, neuronal avalanche, power-law, scaling relation, branching ratio, statistical tests, standardization

## Abstract

The study of the brain criticality hypothesis has been going on for about 20 years, various models and methods have been developed for probing this field, together with large amounts of controversial experimental findings. However, no standardized protocol of analysis has been established so far. Therefore, hoping to make some contributions to standardization of such analysis, we review several available tools used for estimating the criticality of the brain in this paper.

## 1. Introduction

There are quite a few excellent reviews regarding the subject of brain criticality by now (see e.g., Wilting et al., 2018; Wilting and Priesemann, 2019a; Plenz et al., 2021; Zeraati et al., 2021). But the field is full of controversies and clearly lacks a standardization. In order to make all the results more unified, a standardization of the tools used for analyzing may be of priority. Here, we review several existing tools for estimating the brain's criticality as a small part of such an attempt.

The subject of brain criticality is closely related to neuronal avalanches. However, the definition of experimental avalanches varies among research due to different recording techniques. A detailed illustration can be found in Girardi-Schappo (2021). Nevertheless, when dealing with discrete time series, a suitable bin size (see Levina and Priesemann, 2017) may be used to extract all the avalanches as long as there exists a clear separation of time scales, namely the time of quiescence between avalanches is much longer compared to the duration of any single avalanche. Theoretical models often bear an intrinsic separation of time scales, thus defining their avalanches is relatively easy and should be standardized. Real experimental data are, however, much more complicated. For example, in reality, the sample size is always very large, and in such a regime, a clear separation of time scales is always not available, since at each time bin, the probability that at least one among all recorded neurons is active can be extremely high, leading to one single avalanche that may never terminate. Yet in fact, in most studies, avalanches are defined as consecutive time bins with at least one active site. Thus, much more work should be devoted to making the definition of avalanches standardized and applicable to different cases. Until then, drawing any conclusion may never be fully convincing.

Besides the definition of avalanches, the analysis process of avalanches is of equal importance, so efforts should be taken to make this process as rigorous and standardized as possible. By far, many methods have been developed for such analysis, and we do not intend to exhaust them all. In the following sections, we review several important tools commonly used to testify the criticality hypothesis in the brain, including the power-law fitting, crackling noise scaling relation, shape collapsing, and branching ratio, hoping to make some contributions to the standardization of avalanche analysis. Of course, a suitable definition of avalanches is presumed.

## 2. Power-Law Fitting

Power-law fitting is essential in most works searching for evidence of brain criticality, but many did not apply rigorous tests to the fitting results. The commonly used way of fitting is by using the maximum likelihood estimation (MLE) method which has many desirable properties (see e.g., Casella and Berger, 2002). But, simply fitting power-law distributions by plain MLE has several drawbacks (see e.g., Goldstein et al., 2004; Bauke, 2007; White et al., 2008; Clauset et al., 2009). Clauset et al. (2009) developed a more rigorous method of fitting untruncated power-law distribution by combining MLE and the Kolmogorov–Smirnov (KS) goodness-of-fit test, allowing statistical evaluation of the fitting results. Approximately, they first derived a distribution of KS values by using synthetic power-law surrogates, which were then compared to the KS value of the empirical data, and the proportion of sample distributions with KS values larger than the KS value between the empirical data and the model distribution gives the *p*-value of rejecting the power-law fit hypothesis. Later Deluca and Corral extended Clauset et al.'s work, making the fitting available for doubly truncated (continuous) power-law distributions (Deluca and Corral, 2013, see Corral and González, 2019 for illustration).

Marshall et al. (2016) developed the Matlab Neural Complexity and Criticality (NCC) Toolbox for doing neuronal avalanche analysis, which included an automated MLE fitting routine based on the method developed by Deluca and Corral, where the fitting range (i.e., from a minimum cutoff to a maximum cutoff) was found automatically instead of being manually determined, detailed illustrations can be found in their paper. They extended previously used MLE techniques to doubly truncated discrete power-law distributions that are more appropriate for describing empirical data which have maximum cutoffs typically caused by finite size effects.

But as pointed out by Corral and González (2019), their method has two important drawbacks, i.e., lacking the estimation of the uncertainty of the minimum and maximum cutoffs and underestimating the uncertainty in the obtained power-law exponent. They provided a solution to tackle these problems, i.e., taking bootstrap resamplings of the original data (Good, 2006) and repeating the fitting procedure with them to obtain distributions for cutoffs and the power-law exponent from which the uncertainty may be estimated. In the meantime, studying the dependence of the fitted power-law exponent on both cutoffs should be a good complementation (Baró and Vives, 2012). Thanks to Serra-Peralta et al. (2022), a Python version of this fitting method is available, and the source codes can be found on Github.

Another available tool is provided by Destexhe and Touboul (2021a), who used a similar MLE-based method for fitting power-laws truncated to a minimum cutoff. The fit was validated by the Akaike information criterion (AIC, see Akaike, 1974), which uses the maximum likelihood value $\hat{L}$ of models


$$AIC=2k-2\log\left(\hat{L}\right)+\frac{2k^{2}+2k}{N-k-1},$$

and the model with smaller AIC value is a relatively better fit for a given data set. However, when using this method for doubly truncated data, a maximum needs to be specified manually. Nevertheless, utilizing the AIC principle may be a good complement to the methods discussed above.

Actually, very similar to the AIC principle, Alstott et al. (2014) tested whether the power-law distribution is the best descriptor of the data compared to alternative heavy-tailed distributions, e.g., the exponential or log-normal distribution, by calculating the log-likelihood ratio (LLR). The underlying reason for doing LLR tests are that real world systems have all kinds of noises, and so few empirical phenomena could be expected to follow a power-law with the perfection of a theoretical distribution, especially in the large sample size regime where even small deviations from a perfect power-law would lead to the rejection of the power-law hypothesis by using the fitting methods discussed above. There is an existing toolbox for doing LLR tests: the power-law Python package, whose descriptions and sources can be found in Alstott et al. (2014).

Additionally, before all fitting procedures, enough number of avalanches should be generated to make the results more accurate, since small samples may cause strong biases. Meanwhile, before fitting the data, a preview of it would be helpful in order to choose a proper fitting procedure. For example, while the NCC toolbox provided a method to automatically find the exponent and the fitting range, it may take an unexpectedly long time to find the range and when it is finally done, it may happen that just a very small part of the data is fitted. Such circumstances may be avoided if the data were plotted first to see how it departs from a pure power-law distribution. However, if the results fitted by these tools are not satisfying, though may be subjective, manually setting the fitting range is always an option, which is just the way used in Carvalho et al. (2021) and many others.

We highly recommend that future research make the best use of these freely available advanced tools for power-law fitting of both empirical data and simulation results, hoping for a much more statistically rigorous and unified results in the future.

## 3. Crackling Noise Scaling Relation

Recent research (see e.g., Ponce-Alvarez et al., 2018; Fontenele et al., 2019; Carvalho et al., 2021; Fosque et al., 2021) have applied the crackling noise scaling relation (Muñoz et al., 1999; Sethna et al., 2001) to estimate the criticality of neural systems, though for which criticisms exist (see Destexhe and Touboul, 2021b). Nevertheless, the scaling relations observed in the spiking activity of mammalian primary visual cortex in both anesthetized (rats and monkey) and freely moving animals (mice) showed striking consistency in a specific activity regime (Fontenele et al., 2019), and the work of Ponce-Alvarez et al. (2018) showed consistent crackling noise scaling relations in whole-brain neuronal activity of zebrafish larvae, making the scaling relation itself interesting enough for further investigation. When using the crackling noise scaling relation, many research often refer to Muñoz et al. (1999) and Sethna et al. (2001), where methods for derivation were proposed, but the relation was not explicitly written out or derived rigorously. There is a simple derivation proposed in Girardi-Schappo (2021), but it seems that the author presumed relation between the size and duration of avalanches, which may lack explanation. Here, we illustrate the derivation of the crackling noise scaling relation briefly based on the work of Sethna et al. (2005). The probability function of having an avalanche of size *S*, duration *T*, long axis *L*, short axis *W*, at disorder *R*, and external field *H* in Sethna et al. (2005) has the following scaling form (see Equation 1 in Sethna et al., 2005, for reference to scaling functions see e.g., Henkel et al., 2008):


(1) $$D(S,T,L,W,R,H)=D_{s}S^{-(\tau +\sigma \upsilon z)}D(\frac{S}{S_{s}}r^{1/\sigma },\frac{T}{T_{s}}r^{z\upsilon },\frac{L}{W},\frac{h}{r^{\beta \delta }}),$$

where τ, σ, υ, β, δ, and *z* are critical exponents, $r=\frac{R-R_{c}}{R_{s}}$, $h=\frac{H-H_{c}}{H_{s}}$, and *D*_*s*_ is a normalization factor. Throwing out non-universal factors, and setting the external field to its critical value, we can write in a more simplified asymptotic scaling form (close to the critical point):


(2) $$D(S,T,r)\sim S^{-(\tau +\sigma \upsilon z)}D(Sr^{1/\sigma },Tr^{z\upsilon }).$$

From (2), we can get the scaling forms for the avalanche sizes


(3) $$\begin{array}{ll} D(S,r) & \sim \int D(S,T,r)dT\\ & \sim \int S^{-(\tau +\sigma \upsilon z)}D(Sr^{1/\sigma },Tr^{z\upsilon })dT\\ & \sim \int S^{-\tau }{\left(Sr^{1/\sigma }\right)}^{-\sigma z\upsilon }D(Sr^{1/\sigma },Tr^{z\upsilon })d[Tr^{z\upsilon }]\\ & \sim S^{-\tau }D_{S}(Sr^{1/\sigma }), \end{array}$$

and durations as well


(4) $$\begin{array}{ll} D(T,r) & \sim \int D(S,T,r)dS\\ & \sim \int S^{-(\tau +\sigma \upsilon z)}D(Sr^{1/\sigma },Tr^{z\upsilon })dS\\ & \sim \int r^{\frac{-1+\tau +\sigma z\upsilon }{\sigma }}{\left(Sr^{1/\sigma }\right)}^{-\tau -\sigma z\upsilon }D(Sr^{1/\sigma },Tr^{z\upsilon })d[Sr^{1/\sigma }]\\ & \sim T^{-\frac{-1+\tau +\sigma z\upsilon }{\sigma z\upsilon }}\int {\left(Tr^{z\upsilon }\right)}^{\frac{-1+\tau +\sigma z\upsilon }{\sigma z\upsilon }}\\ & {\left(Sr^{1/\sigma }\right)}^{-\tau -\sigma z\upsilon }D(Sr^{1/\sigma },Tr^{z\upsilon })d[Sr^{1/\sigma }]\\ & \sim T^{-\tau_{t}}D_{T}(Tr^{z\upsilon }), \end{array}$$

where $\tau_{t}=\frac{-1+\tau +\sigma z\upsilon }{\sigma z\upsilon }$, $D_{S}$ and $D_{T}$ are some scaling functions. Meanwhile, given avalanche duration T, we have the following average avalanche size


(5) $$\begin{array}{ll} \langle S\rangle (T,r) & \sim \int SD(S|T,r)dS\\ & \sim \int S\frac{D(S,T,r)}{D(T,r)}dS\\ & \sim \int \frac{S^{1-(\tau +\sigma \upsilon z)}D(Sr^{1/\sigma },Tr^{z\upsilon })}{T^{-\frac{-1+\tau +\sigma \upsilon z}{\sigma z\upsilon }}D_{T}(Tr^{z\upsilon })}dS\\ & \sim \int T^{\frac{1}{\sigma \upsilon z}}{\left(Tr^{z\upsilon }\right)}^{\frac{-2+\tau +\sigma \upsilon z}{\sigma \upsilon z}}{\left(Sr^{1/\sigma }\right)}^{1-(\tau +\sigma \upsilon z)}\\ & \frac{D(Sr^{1/\sigma },Tr^{z\upsilon })}{D_{T}(Tr^{z\upsilon })}d[Sr^{1/\sigma }]\\ & \sim T^{a}D^{\prime }(Tr^{z\upsilon }), \end{array}$$

where again *D*′ is some scaling function, and $a=\frac{1}{\sigma \upsilon z}$. Combining (3)−(5), we get the crackling scaling relation:


$$\frac{\tau_{t}-1}{\tau -1}=a.$$

Whether the above equation holds or not may be tested by using the two-sample *t*-test (see e.g., Ponce-Alvarez et al., 2018; Destexhe and Touboul, 2021b), a statistical method for testing whether the unknown population means of two groups are equal or not. Obviously, such a test relies on the estimation of power-law fitting exponents, thus care must be taken when drawing conclusions from the results.

Note also that the exponents can be obtained using different methods, and they should be consistent across all reliable methods. Taking the above exponent $\frac{1}{\sigma \upsilon z}$ as an example, Ponce-Alvarez et al. (2018) showed that the power spectral density (PSD) of the time courses of neuronal avalanches decays also with exponent $\frac{1}{\sigma \upsilon z}$, thus one can estimate it from the relation (5), through the decay of the avalanche PSD, and through other possible ways like the scaling shape collapse discussed in the next section. The estimated $\frac{1}{\sigma \upsilon z}$ should be consistent across these different ways of estimating the exponent, and the same should apply to estimating other exponents.

Another point is that the scaling relation is one way to reveal some spatiotemporal statistics of the data, and for neuronal avalanche analysis uncovering the true spatiotemporal statistics of the system, results should be compared to surrogate datasets to avoid false conclusions. For instance, one can use randomized surrogates that destroy the correlations or time-shuffled surrogates that destroy the temporal organization of the data while preserving the spatial correlations (see e.g., Ponce-Alvarez et al., 2018).

## 4. Scaling Shape Collapse

Apart from power-law distributions and scaling relations, the existence of universal scaling functions which capture the dynamics of systems at different scales can be used as a further signature of criticality.

Universal scaling functions are one of the most important predictions of universality (see Sethna et al., 2001). The time history of the average size over all avalanches of fixed duration *T*, denoted as 〈*V*〉(*T, t*), is one example of universal scaling functions and has the following scaling form


$$\langle V\rangle \left(T,t\right)=T^{b}V\left(t/T\right),$$

where *b* is some scaling exponent. If we plot *T*^−*b*^〈*V*〉(*T, t*) vs. scaled time *t*/*T*, then we are supposed to observe what is called scaling shape collapse, a simple way to check the scaling hypothesis and measure the universal scaling function. However, quantifying the quality of shape collapses is difficult and can be very subjective by just looking at the plotted results. Included in their NCC toolbox, Marshall et al. (2016) developed a shape collapse algorithm that did not intend to determine if a particular data set exhibits shape collapse, but automatically found the scaling parameter that produced the best possible collapse instead. Specifically, it used linear interpolation to each avalanche at a number of points along the scaled duration *t*/*T* and then calculated the variance among all the avalanche profiles at the interpolated points. The final fitted scaling exponent is the one that minimized the shape collapse error among a range of possible exponent values, and this whole analysis can be quantitated, for details see Marshall et al. (2016). Compared to the former shape collapse analysis method (see e.g., Friedman et al., 2012), such shape collapse method takes much more avalanches into account and is automated and quantitative, though not able to determine whether a given data set exhibits shape collapses.

Attempts have been made to directly quantify the quality of shape collapses by Shaukat and Thivierge (2016), where a complicated method based on functional data analysis was proposed. Their method first smoothed avalanches with a Fourier basis, followed by rescaling using a time-warping function, and finally employed an F-test combined with a bootstrap permutation to determine if avalanches collapse together in a statistically reliable way. Although seemingly more statistically rigorous, this method also seemed not able to measure the scaling parameter or the universal scaling function. Therefore, the analysis of scaling shape collapse obviously requires more future research, and for now, these existing well-established methods should be used when analyzing scaling shape collapse to avoid being subjective.

## 5. Branching Ratio

Apart from studying distribution functions of avalanches, analyzing the branching ratio of events which describes activity propagation is a complementary approach to test if a system is critical or not, where the branching ratio is defined as the average number of descendants per ancestor (de Carvalho and Prado, 2000, for reference to the branching process see Harris et al., 1963). A branching ratio being smaller than, equal to or larger than 1 would imply a subcritical, critical or supercritical system, respectively. The fundamental work established by Beggs and Plenz (2003) showed that the branching ratio of their experimental data, defined as the ratio of the average number of electrodes activated in two consecutive time bins, was close to 1, indicating a critical branching process as the mechanism behind the power-law distributions in cortical networks. Such a fascinating scenario inspired a great amount of work in the field, and many research studies also applied the branching ratio as one way of defining the criticality of neural systems (see e.g., Haldeman and Beggs, 2005; Beggs, 2008; Williams-Garcia et al., 2014; Timme et al., 2016).

However, the conventional estimator of the branching ratio using linear regression can be strongly biased under subsampling (Wilting and Priesemann, 2018), which unfortunately happens to be the case in real experiments. Luckily, Wilting and Priesemann (2018) derived a novel estimator (called MR estimator) based on multistep regression, which was analytically proved to be consistent under subsampling. They showed that the branching ratio was close to but slightly smaller than 1 and proposed that the cortex may be operating in a reverberating regime. Short after that Wilting et al. (2018) proposed that operating in a reverberating regime may enable the cortex to rapidly tune itself according to various task requirements. Such an opinion is very interesting and bears its own reasoning and makes the estimation of the branching ratio worth more considering in future research.

Now there is a python toolbox developed by Spitzner et al. (2021), and future research estimating the branching ratio should utilize these novel tools as much as possible.

## 6. Discussion

The current opinions regarding the subject of brain criticality vary among researchers, it remains unclear whether or not the brain possesses self-organized criticality, quasicriticality, supercriticality, or subcriticality, or rather it operates in a reverberating regime, or it may have many critical states, or perhaps, it is so complicated that its dynamics could not be well explained by any existing theory (for reviews see e.g., Priesemann, 2014; Williams-Garcia et al., 2014; Costa et al., 2017; Wilting et al., 2018; Wilting and Priesemann, 2019b; Fosque et al., 2021; Gross, 2021; Plenz et al., 2021; Zeraati et al., 2021). Nevertheless, probing underlying mechanisms of the complex brain dynamics is rather intriguing and needless to say, important. Several tools as part of such an attempt have been reviewed in this article. But as pointed out by Destexhe and Touboul (2021b), there is currently no clear evidence that the crackling noise scaling relation is a sufficient or necessary condition for the criticality of the brain, and the same happens to other methods used for determining brain's criticality as well. Future research should try to utilize the available statistically reliable tools for analyzing both experimental and theoretical results so as to make them less controversial and more rigorous. Meanwhile, though full of challenges, developing new more advanced reliable tools is of essence to eventually reaching unified and convincing conclusions.

## Author Contributions

The author confirms being the sole contributor to this work and has approved it for publication.

## Funding

CY was supported by the National Natural Science Foundation of China (11671354).

## Conflict of Interest

The author declares that the research was conducted in the absence of any commercial or financial relationships that could be construed as a potential conflict of interest.

## Publisher's Note

All claims expressed in this article are solely those of the authors and do not necessarily represent those of their affiliated organizations, or those of the publisher, the editors and the reviewers. Any product that may be evaluated in this article, or claim that may be made by its manufacturer, is not guaranteed or endorsed by the publisher.
